# Selenium-L-methionine modulates radiation injury and Duox1 and Duox2 upregulation in rat’s heart tissues

**DOI:** 10.15171/jcvtr.2019.21

**Published:** 2019-06-27

**Authors:** Sedighe Kolivand, Peyman Amini, Hana Saffar, Saeed Rezapoor, Masoud Najafi, Elahe Motevaseli, Farzad Nouruzi, Dheyauldeen Shabeeb, Ahmed Eleojo Musa

**Affiliations:** ^1^Department of Medical Biotechnology, School of Advanced Technologies in Medicine, Tehran University of Medical Sciences, Tehran, Iran; ^2^Department of Radiology, Faculty of Paramedical, Tehran University of Medical Sciences, Tehran, Iran; ^3^Clinical and Anatomical Pathologist at Tehran University of Medical Science, Imam Khomeini Hospital Complex, Tehran, Iran; ^4^Radiology and Nuclear Medicine Department, School of Paramedical Sciences, Kermanshah University of Medical Sciences, Kermanshah, Iran; ^5^Department of Molecular Medicine, School of Advanced Technologies in Medicine, Tehran University of Medical Sciences, Tehran, Iran; ^6^Department of Medical Radiation Engineering, Science and Research Branch, Islamic Azad University, Tehran, Iran; ^7^Department of Physiology, College of Medicine, University of Misan, Misan, Iraq; ^8^Research Center for Molecular and Cellular Imaging, Tehran University of Medical Sciences (International Campus), Tehran, Iran

**Keywords:** Radiation, Selenium-L-Methionine, Heart, IL-4, Duox1, Duox2

## Abstract

***Introduction:*** Redox interactions play a key role in radiation injury including heart diseases. In present study, we aimed to detect the possible protective role of selenium-L-methionine on infiltration of immune cells and Duox1&2 upregulation in rat’s heart tissues.

***Methods:*** In this study, 20 rats were divided into 4 groups (5 rats in each) namely: irradiation; irradiation plus Selenium-L-methionine; control; and Selenium-L-methionine treatment. Irradiation (15 Gy to chest) was performed using a cobalt-60 gamma ray source while 4 mg/kg of selenium-L-methionine was administered intraperitoneally. Ten weeks after irradiation, rats were sacrificed for detection of IL-4 and IL-13 cytokines, infiltration of macrophages and lymphocytes as well as the expressions of IL4Ra1, Duox1, IL13Ra2 and Duox2.

***Results:*** Results showed an increase in the level of IL-4 as well as the expressions of IL4Ra1, Duox1 and Duox2. Similarly, there was an increase in the infiltration of lymphocytes and macrophages. There was significant attenuation of all these changes following treatment with selenium-L-methionine.

***Conclusion:*** Selenium-L-methionine has the potential to protect heart tissues against radiation injury. Downregulation of pro-oxidant genes and modulation of some cytokines such as IL-4 are involved in the radioprotective effect of selenium-L-methionine on heart tissues.

## Introduction

Cardiovascular disorders are among the most common threats for people exposed to ionizing radiation.^1^ Some epidemiological reports show that people who were exposed to ionizing radiation during the Hiroshima and Nagasaki incidents had an increased risk of death as a result of heart diseases.^2^ This issue was also confirmed for the Chernobyl disaster.^3^ However, a major concern for radiation-induced cardiovascular disorders is related to the side effects from radiotherapy for cancer patients. Studies have shown a significant increase in heart diseases for patients with left breast cancer compared to those with right breast cancer.^4^ Increased incidence of heart diseases was observed for patients who underwent radiotherapy with the heart exposed to whole body irradiation (for bone marrow transplantation) or Hodgkin’s disease.^5^ The American Society of Clinical Oncology reported a 30% increase in the incidence of heart diseases 10 years after treatment.^6^ It has been proposed that there is a linear relation between the received radiation doses to heart tissue and the probability of cardiovascular disorders.

The most obvious manifestations of cardiac injury following exposure to radiation include fibrosis, inflammatory reactions (such as chronic pericarditis and myocarditis) and atherosclerosis.^5^ Infiltration of inflammatory cells such as lymphocytes, macrophages and mast cells is a common manifestation of cardiac injury induced by ionizing radiation.^7^ It seems that accumulation of inflammatory cells induces chronic oxidative stress via increasing pro-inflammatory and pro-fibrotic cytokines as well as upregulation genes involved in reduction/oxidation reactions in a positive feedback. Previous studies proposed the increased levels of interleukin-1 (IL-1) and transforming growth factor beta (TGF-β) in heart tissue following exposure to radiation.^8^ In recent years, it has been proposed that upregulation of some pro-oxidant enzymes such as NADPH oxidase (NOX1-5) and dual oxidase 1&2 (Duox1&2), and COX-2 play a key role in chronic oxidative damage following exposure to radiation.^9,10^ IL-4 and IL-13 are two important pro-fibrosis cytokines that mediate radiation-induced fibrosis and chronic oxidative stress. IL-4 and IL-13 are able to potentiate infiltration and maintenance of macrophages to irradiated tissues, leading to chronic inflammation and oxidative stress.^11,12^ Molecular studies have shown that IL-13 has ability to induce upregulation of Duox1. However, IL-4 can induce upregulation of both Duox1 and Duox2, thus causes generation of reactive oxygen species (ROS). Upregulation of both Duox1 and Duox2 has been shown following exposure to radiation.^13^ Furthermore, inhibition of IL-4 and IL-13, as well as Duox1 and Duox2 have been proposed for reducing radiation-induced normal tissues injury.^13,14^

Several natural and chemical radioprotectors have been tried out in experimental studies for amelioration of radiation toxicity in the heart.^15^ Amifostine, an FDA approved drug for alleviation of xerostomia for patients with head and neck cancers, has also shown some radioprotective properties for the heart.^16,17^ In some experimental studies, it was observed that treatment with amifostine showed ability to ameliorate interstitial and vascular fibrosis as well as myocyte necrosis, but could not reduce inflammation in heart tissues.^17,18^ Toxicity of amifostine may cause some problems such as nausea and low blood pressure in patients. In recent years, natural and low toxic agents have been developed for protection against radiation toxicity. Selenium-L-methionine is a potent antioxidant comprising selenium and methionine. It is involved in cellular antioxidant defense through the stimulation of glutathione and other antioxidant enzymes. Methionine acts as an antioxidant within cells.^19^ In present study, we aimed to examine the possible radioprotective effect of selenium-L-methionine in rat’s heart tissues. We defined the protective effect if it is against infiltration of inflammatory cells as well as upregulation of Duox1 and Duox2 signaling pathways.

## Materials and Methods

### Treatment and irradiation

Selenium-L-methionine powder, purchased from Sigma Aldrich (USA), was dissolved in distilled water to give a concentration of 0.8 mg/mL. Each rat received an intraperitoneal (IP) injection of 4 mg/kg (1 mL) of the solution containing selenium-L-methionine. Selenium-L-methionine injected for 6 consecutive days starting one day before irradiation and continued for 4 days after day of irradiation. In the day of irradiation, rats received IP injection of selenium-L-methionine, 30 minutes before irradiation. This dose of Selenium-L-methionine was chosen based on a previous study as a non-toxic dose.^20^ Furthermore, this dose of selenium has been shown to ameliorate deleterious effects of radiation in normal tissues.^21^ On the day of irradiation, rats were anesthetized using ketamine (80 mg/kg) and xylazine (5 mg/kg). Based on our previous study,^7^ using a field size of 25×5 cm^2^ with rats placed supine, irradiation was performed using a Cobalt-60 (^60^Co) gamma ray source with a dose of 15 Gy to their chests at a dose rate of 109 cGy/min and 60 cm source to skin distance (SSD). This dose of radiation was based on a previous study by Ibis et al. for evaluating the radioprotective effect of amifostine and L-carnitine on rat’s heart tissues.^22^

### Experimental design

Twenty male healthy rats were purchased from Razi institute, Tehran, Iran. All rats were kept under standard conditions of humidity (55%) and temperature (25^0^C), as well as constant light (5 AM-5 PM) and dark (5 PM-5 AM) cycle. The rats were divided into 4 groups (5 rats in each) namely; 1: local irradiation of chest area without treatment; 2: local irradiation of chest area with treatment with selenium-L-methionine; 3: treatment with selenium-L-methionine alone without irradiation; 4: control without any irradiation or treatment with selenium-L-methionine. The sample size for each group was chosen based on previous study.^14^ 10 weeks after irradiation, all rats were sacrificed and their heart tissues removed. The choice of this time was also based on our previous study.^7^ The lower parts of their heart tissues were fixed in 10% formalin for pathological evaluation while their upper parts were frozen for detection of gene expression as well as IL-4 and IL-13 levels.

### Histopathological evaluation

After fixation with formalin buffer, heart tissues were embedded in paraffin and cut into 5 micron sections. Two sections from each sample were placed on slides for staining. One section was stained with *Masson’s Trichrome* (MTC) for detection of collagen deposition while the other section was stained with hematoxylin and eosin (H&E) for the detection of morphological changes such as infiltration of lymphocytes and macrophages. All slides were studied with the aid of a light microscope at 100x magnification by a pathologist at Imam Khomeini hospital, Tehran University of Medical Sciences, Tehran, Iran.

### IL-4 and IL-13 detection

The levels of IL-4 and IL-13 were detected using ELISA kit (ZellBio, Germany) based on the manufacturer’s instruction. Results of the samples were normalized to standard curve and reported as pictogram per milliliter (pg/mL).

### Real-time PCR

Total RNA was extracted using RNX^+^ solution (Sinacolon, Iran) while cDNA was synthetized using cDNA synthesis kit (Takara, Japan) by a thermocycler instrument. The RNA concentration was determined using NanoDrop while its purity was checked using electrophoresis. Real-time polymerase chain reaction (PCR) was done using Applied Biosystems and cyber green master mix (Takara, Japan). The primer sequences used for real-time PCR are shown in Table 1.

**Table 1:** Forward and Reverse Sequences of Primers

**Gene**	**Forward sequence**	**Reverse sequence**	**Product length**
*IL-4R1*	GAGTGAGTGGAGTCCCAGCATC	GCTGAAGTAACAGGTCAGGC	127bp
*IL-13Ra2*	TCGTGTTAGCGGATGGGGAT	GCCTGGAAGCCTGGATCTCTA	120bp
*Duox1*	AAGAAAGGAAGCATCAACACCC	ACCAGGGCAGTCAGGAAGAT	147bp
*Duox2*	AGTCTCATTCCTCACCCGGA	GTAACACACACGATGTGGCG	165bp
*GAPDH*	AGTGCCAGCCTCGTCTCATA	ATGAAGGGGTCGTTGATGGC	133bp

For real-time PCR, each sample was run in duplicate. At first, the mean CT for each target gene and internal control gene were calculated. The difference between these means was given as ΔCT, while ΔΔCT was calculated as the difference between the mean ΔCT. 2^-RΔΔCT^ was used to achieve relative fold changes for IL4ra1, IL13ra2, Duox1 and Duox2 after normalization to GAPDH gene.

### Statistical analysis

All statically analyses were done using SPSS software version 16 (IBM, Chicago, USA). T-Test was used to analyze real-time PCR results while ELISA results were analyzed using one-way ANOVA. The differences between groups were considered statistically significant for *P* value < 0.05.

## Results

### Histopathological evaluation

**Figure 1:**
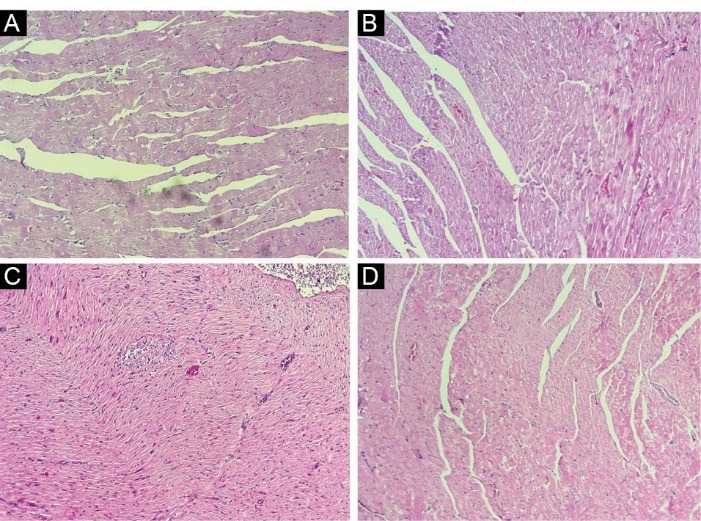
Radioprotective effect of Selenium-L-methionine on histological changes in rat’s heart tissues. (A) Control: heart tissues show normal appearance; (B) Selenium-L-methionine treatment: tissues were similar to control; (C) Radiation: infiltration of lymphocytes and macrophages were remarkable (block dots); (D) Radiation plus selenium-L-methionine: tissues were similar to A and B (H&E staining ×100).

Radiation could significantly increase the infiltration of macrophages and lymphocytes. However, there were no signs of edema, vascular damage or fibrosis. Selenium-L-methionine administration before and after irradiation of heart tissues was able to completely reverse the infiltration of both lymphocytes and macrophages (Figure 1).

### ELISA

**Figure 2:**
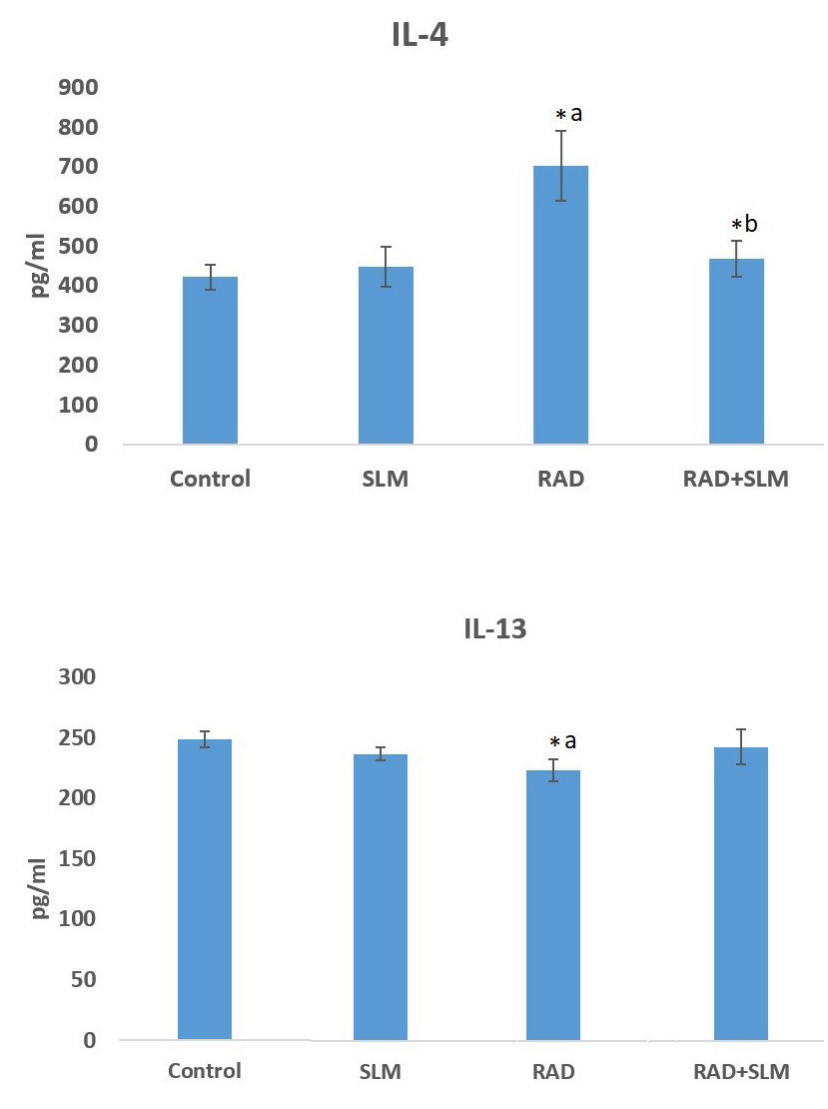
Changes in the levels of IL-4 and IL-13 following irradiation of rat’s heart tissues and treatment with selenium-L-methionine. RAD=irradiated group, SLM= treatment with selenium-L-methionine. (*P < 0.05, a: significant compared to control; b: significant compared to radiation group).

The concentrations of both IL-4 and IL-13 have a direct relation with the blur product of ELISA assay due to the effects of biotinylated antibody and streptavidin-HRP conjugate. As shown in Figure 2, exposure to ionizing gamma rays led to an increase in the level of IL-4 compared to control group (*P* < 0.05). However, there was a significant reduction in the level of IL-13 after irradiation (*P* < 0.05). Treatment with selenium-L-methionine before and after exposure to radiation could significantly reduce the level of IL-4, but could not change the level of IL-13 (*P* < 0.05) (Figure 2).

### Real-time PCR

**Figure 3:**
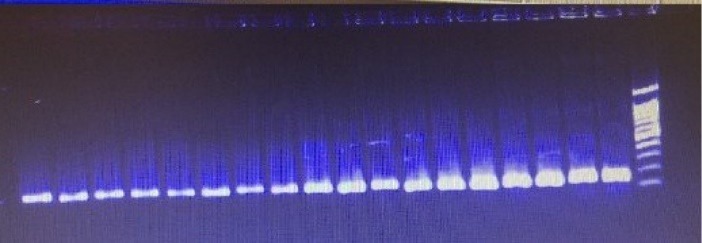
Results of electrophoresis gel following the extraction of total RNA.

RNA extraction results showed appropriate concentration and also confirmed the extraction protocol (Figure 3). Absorbance ratio results for our samples showed that the 260 nm/A280 nm ratio was more than 1.9.

**Figure 4:**
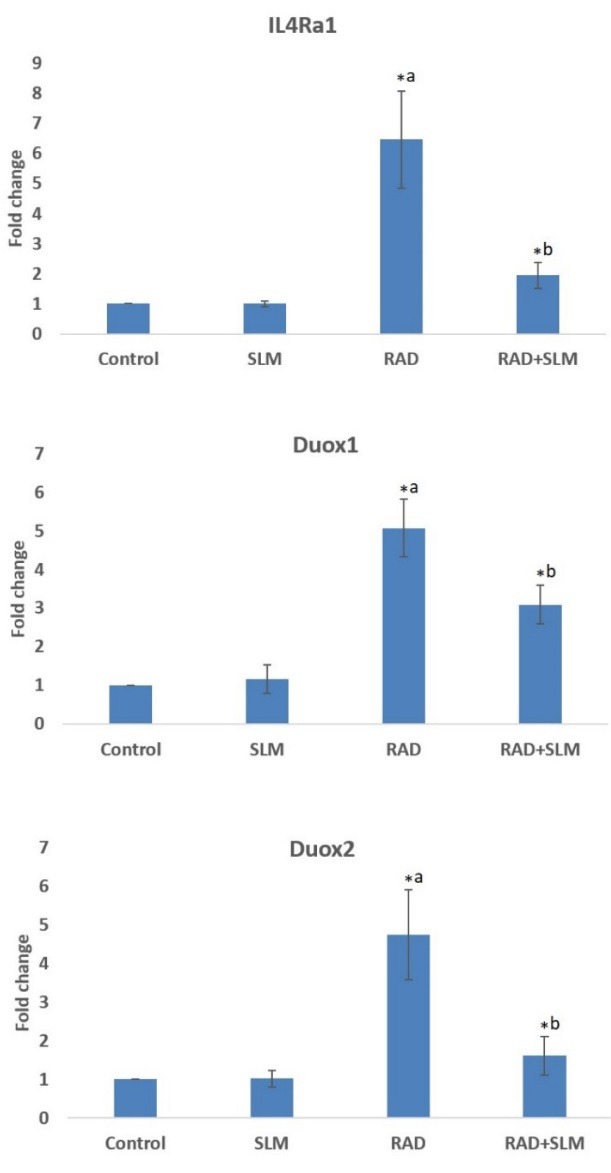
Changes in the expressions of IL4Ra1, Duox1 and Duox2 following irradiation of rat’s heart tissues and treatment with selenium-L-methionine. Selenium-L-methionine treatment group or radiation group was compared to control group, while selenium-L-methionine plus radiation group was compared with radiation group. RAD=irradiated group, SLM= treatment with selenium-L-methionine. (* *P* < 0.05, a: significant compared to control; b: significant compared to radiation group).

Real-time PCR results showed that exposure of rat’s heart tissues led to a significant increase in the expression of IL4Ra1 (6.47 ± 1.61 fold) compared to control (*P* < 0.05). For the radiation plus selenium-L-methionine group, a significant attenuation in the expression of IL4Ra1 compared to radiation group was observed (1.95 ± 0.43 fold compared to control). However, treatment with only selenium-L-methionine did not cause any change in the expression of this gene (1 ± 0.08 fold compared to control). Results also showed no detectable expression of IL13Ra2 in the examined heart tissues. The expression of Duox1 was increased following irradiation of heart tissues (5.07 ± 0.74 fold compared to control), while its expression reduced when rats were treated with selenium-L-methionine (3.09 ± 0.50 fold compared to control) (*P* < 0.05). The expression of Duox1 did not change following treatment with only selenium-L-methionine (1.15 ± 0.36 fold compared to control). Furthermore, the expression of Duox2 was increased following irradiation of rat’s heart tissues (4.75 ± 1.16 fold compared to control). However, its expression was attenuated when rats were treated with selenium-L-methionine before and after irradiation (1.61 ± 0.50 fold compared to control) (*P* < 0.05) (Figure 4).

## Discussion

As our results have shown, irradiation of rat’s heart tissues led to increased level of IL-4. This was associated with upregulation of the expression of its receptor IL4Ra1. As earlier mentioned, IL-4, through the stimulation of its receptor, can cause chronic production of H_2_O_2_ and oxidative injury via Duox1 and Duox2 stimulation. Our results showed a significant upregulation of Duox2 in heart cells. Interestingly, the expression of Duox1 was upregulated by more than 5-fold. However, we could not detect the expression of IL13Ra2. These changes were associated with increased infiltration of inflammatory cells such as lymphocytes and macrophages. Administering selenium-L-methionine 24 h before irradiation and four consecutive days after irradiation, could significantly reverse infiltration of lymphocytes and macrophages. In addition, treatment with selenium-L-methionine attenuated the level of IL-4 and its downstream genes including IL4Ra1, Duox1 and Duox2. The expression of Duox1 reduced from 5-fold to 3-fold following selenium-L-methionine treatment.

So far, several studies have been conducted to detect the mechanisms involved in radiation-induced cardiovascular injury. Epidemiological and prospective studies have shown that people who were exposed to total body irradiation or radiotherapy for left breast cancer have higher risks for various signs of cardiovascular disorders such as ischemia, atherosclerosis, carotid and coronary diseases as well as heart attack.^23^ Experimental studies have been conducted to illustrate the molecular mechanisms involved in radiation-induced heart diseases. Studies have shown that exposure to radiation leads to chronic increase in the level of some cytokines such as IL-1 and TGF-β in heart tissue.^24^ The increased serum level of TGF-β has been confirmed for Chernobyl disaster survivors 20 years after exposure.^25^ Recent studies have proposed some targets for mitigation of radiation injury in crucial organs such as the heart. Studies have proposed that in addition to radiation-induced ROS, changes in the metabolism of oxygen and nitrogen play a key role in both acute and late effects of radiation in normal tissues. Free radical production by reduction/oxidation mediators such as mitochondria, NADPH oxidase enzymes, COX-2, iNOS and renin-angiotensin system are well known mechanisms for chronic oxidative stress following exposure to radiation.^26^ Duox1 and Duox2 are subfamilies of NADPH oxidase that mediate chronic production of ROS and other consequences following exposure to radiation.^13^ The results of this study suggest that upregulation of dual oxidase genes’ expression may be involved in chronic redox interactions following exposure of heart to ionizing radiation. Hence, attenuation of these genes may be proposed for mitigation of radiation-induced cardiac injury.

Some studies have shown that selenium-L-methionine has a potent protective effect against toxic effects of ionizing radiation.^27-29^ Treatment with selenium-L-methionine can alleviate the formation of micronuclei in the bone marrow cells of rats. Furthermore, it has shown ability to ameliorate radiation toxicity in the kidneys of irradiated rats.^30^ The combinations of selenium-L-methionine with some other antioxidants such as co-enzyme Q-10, N-acetyl cysteine, alpha-lipoic acid, vitamin E and sodium ascorbate have shown abilities to potently protect and mitigate radiation toxicity and oxidative injury in the gastrointestinal and bone marrows of irradiated mice.^31^ In present study, we showed that selenium-L-methionine can be proposed as a radioprotector for heart tissue.

## Conclusion

This study has shown that exposure to ionizing radiation can upregulate the expression of Duox1 and Duox2; two important pro-oxidant enzymes. It is possible that chronic upregulation of these genes may be involved in radiation-induced cardiac injury such as infiltration of macrophages and lymphocytes. Treatment of rats with selenium-L-methionine showed potential to attenuate radiation injury in the heart and also downregulate increased expression of these genes. It is possible that selenium-L-methionine through modulation of the expression of these pro-oxidant enzymes could attenuate radiation toxicity and chronic oxidative injury.

## Ethical approval

This study was accordance with ethical laws provided by Tehran University of Medical Sciences, ethical code: IR.TUMS.VCR.REC.1396.4419.

## Competing interests

All authors declare no competing financial interests exist.

## Funding

Tehran University of Medical Sciences and Health Service supported the study (grant #36668).
